# New ABCC2 rs3740066 and rs2273697 Polymorphisms Identified in a Healthy Colombian Cohort

**DOI:** 10.3390/pharmaceutics10030093

**Published:** 2018-07-17

**Authors:** Rosa Helena Bustos-Cruz, Luis Rafael Martínez, Julio César García, George E. Barreto, Fernando Suárez

**Affiliations:** 1 Evidence-Based Therapeutics Group, Clinical Pharmacology, Universidad de La Sabana, 140013 Chía, Colombia; luismacu@unisabana.edu.co (L.R.M.); Julio.Garcia@unisabana.edu.co (J.C.G.); 2 Departamento de Nutrición y Bioquímica, Facultad de Ciencias, Pontificia Universidad Javeriana, 110231 Bogotá, Colombia; gsampaio@javeriana.edu.co; 3 Instituto de Ciencias Biomédicas, Universidad Autónoma de Chile, 7500912 Santiago, Chile; 4 Instituto de Genética, Facultad de Medicina, Pontificia Universidad Javeriana, Hospital Universitario San Ignacio, 110231 Bogotá, Colombia; fernando.suarez@javeriana.edu.co

**Keywords:** ATP-Binding-cassette (ABC), MRP1 and MRP2, polymorphisms, drug resistance, genetic variability

## Abstract

Multidrug resistance-associated proteins (MRP) 1 and 2 belong to the ABC (ATP-Binding Cassette) transporters. These transport proteins are involved in the removal of various drugs and xenobiotics, as well as in multiple physiological, pathological, and pharmacological processes. There is a strong correlation between different polymorphisms and their clinical implication in resistance to antiepileptic drugs, anticancer, and anti-infective agents. In our study, we evaluated exon regions of MRP1 (ABCC1)/MRP2 (ABCC2) in a Colombian cohort of healthy subjects to determine single nucleotide polymorphisms (SNPs) and to determine the allelic and genomic frequency. Results showed there are SNPs in our population that have been previously reported for both MRP1/ABCC1 (rs200647436, rs200624910, rs150214567) and MRP2/ABCC2 (rs2273697, rs3740066, rs142573385, rs17216212). Additionally, 13 new SNPs were identified. Evidence also shows a significant clinical correlation for polymorphisms rs3740066 and rs2273697 in the transport of multiple drugs, which suggests a genetic variability in regards to that reported in other populations.

## 1. Introduction

The ABC (ATP Binding Cassette or ABC transporters) transporters are formed by a large family of transmembrane proteins that bind ATP and use the energy of ATP hydrolysis to transport various components across the cell membrane [1]. The expulsion of the ABC transporters of the active agent has significant clinical relevance for most drugs used today [2]. A total of 49 genes have been identified for the family of ABC transporters in humans and they have been classified into seven distinct subfamilies, which have been designated by groups of letters while the number of members in parentheses are indicated here: ABCA (12), ABCB (11), ABCC, (13), ABCD (4), ABCE (1), ABCF (3), and ABCG (5). The ABCC subfamily members are also referred to as MRP-ABCC transporters [3,4]. New studies have revealed the role of MRP1 (multidrug resistance-associated protein 1) and MRP2 (Multidrug resistance-associated protein 2) in the transport of drugs and metabolites in multiple physiological, pathological, and pharmacological processes [5,6,7,8,9].

The MRP1/ABCC1 (16p13.1) gene encodes a protein of about 1531 amino acids with a molecular weight of 180–190 KDa [10,11], which functions as a multi-specific transport of organic anions, with glutathione (oxidized), cysteinyl leukotrienes, and aflatoxin B1 activated as substrates. The transport of these conjugates helps the cell remove toxins and protect tissues from damage caused by the expression of mediators of the inflammatory response that controls vascular permeability and smoothens muscle contractions [5]. Likewise, it can carry antineoplastic drugs such as anthracyclines, epipodophyllotoxins, vinca alkaloid, camptothecin, methotrexate, and mitoxantrone [12]. The overexpression of MRP1/ABCC1 has also been shown to allow multidrug resistance to cancer chemotherapy [13]. MRP1/ABCC1 can also carry other drugs such as HIV antiretrovirals, and it is involved in the clinical response to treat this disease [14].

Gene MRP2/ABCC2 (10q24) encodes a protein with 1545 amino acids and a molecular weight of 174 kDa [15]. Unlike MRP1, which is located on the basolateral membrane of the endothelial cells, MRP2/ABCC2 is found in the portion of the apical canalicular membrane of the hepatocyte, the luminal membrane of the small intestine, and the luminal membrane of proximal tubules of the kidney, placenta, and hemato-encephalic barrier [16,17]. Thus, MRP2/ABCC2 reduces the oral absorption, increases renal clearance, and prevents the entry of various drugs to the central nervous system. MRP2/ABCC2 has similar substrates to MRP1 such as the antineoplastic drugs like vinblastine, which appears to contribute to the resistance to the action of anticancer drugs [18,19].

Several drugs are substrates for MRP1, and MRP2 proteins and the polymorphisms associated with each gene affect the response to the treatment and to its toxicity [5] in such a way that the MRP proteins are involved in the success or failure of the treatment of infectious diseases. Table 1 shows the inherent differences in the metabolism, transport, disposal, and toxicity of some anti-infection drugs and their clinical consequence regarding polymorphisms or mutations of the MRP1/ABCC1 and MRP2/ABCC2 genes. The clinical implication is that anti-infection drugs are controlled by polyspecific membrane conveyors expressed in the intestine, liver, kidney, placenta, testicles, blood cells, and cells of the endothelial lineage of brain capillaries that make up the hematoencephalic barrier [20]. The expression and activity of MRPs may also be altered by the presence of certain drugs and disease-related conditions. For example, mRNA levels of MRP2/ABCC2 are reduced by 30% in patients with hepatitis C. Likewise, inhibition and reduction of the expression of MRP2 have suggested the induction of hyperbilirubinemia. Thus, patients with MRP2/ABCC2 deficiency, e.g., those with Dubin-Johnson syndrome, are particularly susceptible to undergoing clinical complications derived from the metabolism of bilirubin and the hepatic metabolism of drugs [20].

Some previous studies report that certain single nucleotide polymorphisms (SNPs) do not correlate with mRNA levels for both MRP1/ABCC1 as MRP2/ABCC2 [21] and that their presence makes no difference in the transport of organic anion [22], while other research has shown that a significant reduction in transportation and increased sensitivity to doxorubicin occurs in the presence of specific SNPs [12]. The presence of particular polymorphisms of MRP2/ABCC2 may cause Dubin-Johnson syndrome [23,24]. Specific polymorphisms have been recently associated to the resistance to antiepileptic drugs in Asian [25] and Caucasian populations [26] for MRP2/ABCC2, which might indicate a certain degree of genotype-phenotype correlation, stratified by type of the SNPs and kind of drug. Based on the importance of the SNPs for the function of MRP1/ABCC1 and MRP2/ABCC2, the frequency of polymorphisms in the genes of interest in the local population needs to be described in order to establish the parameters required to develop personalized medicine, aimed at the prevention of adverse events and increasing treatment success.

## 2. Materials and Methods

### 2.1. Study Population

This is a descriptive study of allelic and genotypic frequencies of polymorphism in a group of exons of the MRP1/ABCC1 and MRP2/ABCC2 genes in a non-random sample population of healthy subjects with no medical history regarding the functions of the genes of interest. This project was approved by the Research Office at the University of La Sabana (MED-165-2013). The study population was a group of inhabitants of the community of the central savannah, in Cundinamarca (Colombia), who were recruited by invitation or by reference from inhabitants of the region, as volunteer subjects in the Hospital of the University of La Sabana. Initially, a total of 29 women and 26 men were included. Each volunteer attended a consultation with a General Practitioner in which a medical history and a complete physical examination were performed. All subjects underwent a battery of paraclinical tests (total blood count, standardized urinalysis, transaminases (SGPT and SGOT), differentiated bilirubin, alkaline phosphatase, serum amylase, fasting-glucose, urea nitrogen, serum creatinine, and quantitative chorionic gonadotropin in the case of women). During the anamnesis, three volunteers were excluded because of the medical history: a volunteer with a history of chondrosarcoma and lung cancer; a subject with a history of hepatitis; and a subject with arterial hypertension. Two other volunteers were excluded as alterations in the results of bilirubin and transaminases were identified. Finally, 50 volunteers who had no medical history of pathologies or alterations in their lab tests were classified under the criteria of healthy volunteers and included. All the subjects provided their informed consent for inclusion before taking part in the study. The project and the informed consent from healthy volunteers were approved by the ethics committee of the University of La Sabana (Acta 251, 26 April 2013). The study was conducted as per the Declaration of Helsinki. According to the results, a decision to inform the volunteers about the details of the findings of this study was made by the research group in a prior meeting.

### 2.2. PCR and DNA Sequencing

A sample of peripheral blood was taken from each subject included in the study. Genomic DNA was extracted from lymphocytes from 50 healthy volunteers using a Purification Kit Wizard^®^ Genomic DNA (Promega A1120, Madison, WI 53711, USA) following the manufacturer’s instructions. DNA quantification was done with a NanoDrop 2000 spectrophotometer (Thermo Scientific, Walhtam, MA, USA) for all samples and an outsourcing service was used for sequencing the exons of interest (Macrogen, Geumcheon-gu, Seoul, Korea).

To accomplish the polymerase chain reactions (PCR) the primers for MRP1/ABCC1 and MRP2/ABCC2 were designed based on previous studies describing the exons of interest related to the presence of polymorphisms in genes that expressed such proteins [23,29]. The primers (Table 2) were designed to amplify the five exons of MRP1 (Ex_2, Ex_10, Ex_16, Ex_17, Ex_23) and five exons of MRP2 (Ex_10, Ex_18, Ex_23, Ex_28, Ex_31). All PCRs were carried out using an MJ Research PTC-225 Peltier Thermal Cycler and a QIAamp DNA Mini Kit (Qiagen, Hilden, Germany) and the Dr. Max DNA Polymerase (Doctor Protein INC, Seoul, Korea) was utilized for the PCR reactions. PCR amplification conditions were as follows: 94 °C for 5 min; 94 °C for 30 s, 30 s of varying temperature, 72 °C for 40 s for 35 cycles; 72 °C for 7 min. PCR products were purified using a Millipore plate MSNU030 (Millipore SAS, Molsheim, France).

### 2.3. Sequencing Analysis

The results were analyzed in five steps: (I) analysis of electropherograms to detect possible conflicts and obtaining the consensus sequence for each exon. The exon sequences reported in NCBI for MRP1/ABCC1 (NG_028268.1) and MRP2/ABCC2 (NG_011798.1) were used as references. The CLC Main Workbench 7.2 (Qiagen Company, Redwood City, USA) was used as a program to perform these analyses. (II) Alignments for each sequence and for each exon were obtained experimentally for both genes, and polymorphisms were then identified along with their genomic location. Samples that did not exhibit a clear and symmetric electropherogram were sequenced bidirectionally again. MUSCLE (Multiple Sequence Comparison by Log-Expectation) (http://www.ebi.ac.uk/Tools/msa/muscle/) was used as a tool to perform the alignments. (III) Genotyping of alleles was performed by analyzing electropherograms of the samples where polymorphisms were located, and the zygosity status for each was determined. (IV) The position of the SNPs identified in genomic DNA was ratified; the tool from the NCBI Variation Viewer (http://www.ncbi.nlm.nih.gov/variation/view/) was used for this purpose by locating the position of each exon, and the SNP was found experimentally and compared to the SNP being referenced. The determination of the genomic position was critical as some polymorphisms which had been found experimentally were located in an intron region. (V) For a description of the identified SNPs, the nomenclature recommendations given by HUGO Gene Nomenclature Committee (Human Genome Gene Nomenclature Committee) were followed. The recommendations and the SNP name building procedure established by the Mutalyzer 2.0.7 (https://mutalyzer.nl/) were followed to appoint the new SNP identified in the study.

### 2.4. Population Analysis of Polymorphisms

The number of homozygous and heterozygous individuals was established for each SNP, as well as the genotype and allele frequencies. Additionally, the Hardy Weinberg (HW) equilibrium for each SNP was calculated according to the formula AA = p^2^, AB = 2pq, BB = q^2^, wherein A and B are the corresponding SNP alleles. To determine whether the genotype frequencies were significantly deviated from what would be expected in the HW balance, the expected genotype frequencies under the HW equilibrium hypothesis were calculated according to the genetic frequencies found in the study. A χ^2^ (chi-square) was applied $C^{2}=\sum \left(\frac{{\left(Observed-expected\right)}^{2}}{expected}\right)$, and a statistical significance of *p* < 0.05 was set. The allele frequencies for each SNP were compared to the allele and genotype frequencies reported by the project 1000 genomes Phase 3. The frequencies found in this study were compared to the global frequencies for each SNP and to the SNP frequencies of a Colombian population that makes part of 1000 Genomes (99 samples of Colombians from Medellín, Colombia). Comparisons were performed using χ^2^ and a statistical significance of *p* < 0.05.

## 3. Results

**Figure 1:**
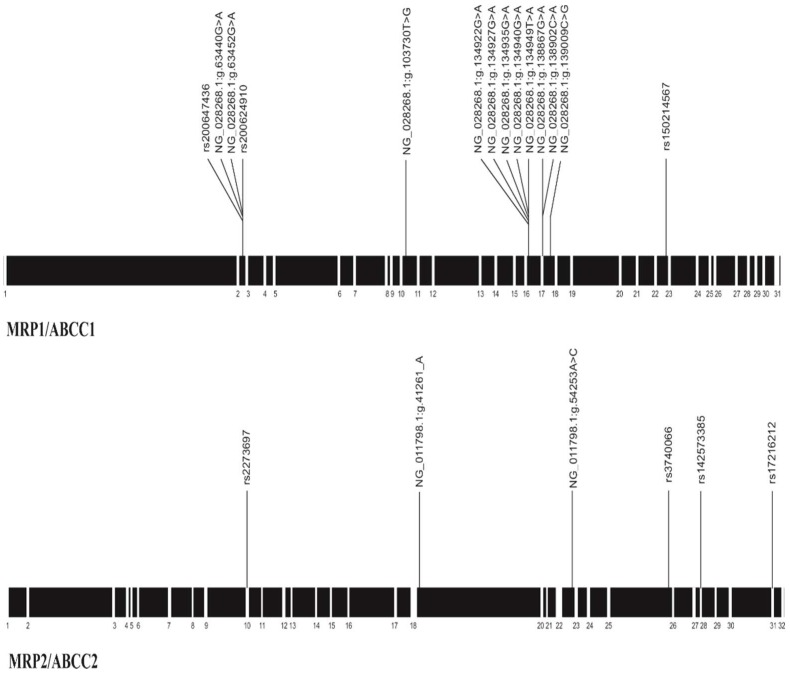
The distribution of polymorphisms identified in this study MRP1/ABCC1 and MPR2/ABCC2. The length of the rectangles is approximately proportional to the extent of exons. The new polymorphisms are highlighted. The black boxes are introns and the white ones, exons.

Fifty healthy volunteers comprised the study population. The electropherograms of the amplicons were analyzed to identify polymorphisms associated with genes expressing MRP1/ABCC1 and MPR2/ABCC2 proteins to determine the allelic and genotypic frequencies of the study population and to compare the allele frequencies to other populations. Figure 1 shows the distribution of SNPs in the exons and introns, on DNA both for the MRP1/ABCC1 protein and for the MPR2/ABCC2 protein. A total of 14 SNPs were identified for MRP1 protein, of which two new polymorphisms were found in exon 17 (NG_028268.1:g.138867G>A y NG_028268.1:g.138902C>A), the remaining 12 were found in regions corresponding to NM_004996.3 (http://www.ncbi.nlm.nih.gov/nuccore/NM_004996.3): between Exon_2 and Exon_3: (rs200647436) NG_028268.1: g.63440G>A (rs 200 624 910) and NG_028268.1: g.63452G>A; between Exon_10 and Exon_11: NG_028268.1: g.103730T>G; between Exon_16 and Exon_17: NG_028268.1: g.134922G>A, NG_028268.1: g.134927G>A, NG_028268.1: g.134935G>A, NG_028268.1: g.134940G>A, and NG_028268.1: g. 134949T>A; between Exon_17 and Exon_18: NG_028268.1: g.139009C>G; between Exon_22 and Exon_23: (rs 150,214,567). The SNP referenced for this protein are rs200647436, rs200624910, and rs150214567, the remaining 11 are not reported previously.

For the MRP2/ABCC2 protein, a total of six SNPs were found and distributed as (Figure 1): Exon_10 (rs2273697), Exon_28 (rs3740066), Exon_31 (rs142573385). Three new ones were found in the introns of the region NM_000392.4 (http://www.ncbi.nlm.nih.gov/nuccore/NM_000392.4) between Exon_18 and Exon_19 (NG_011798.1: g.41261_A) after Exon_23 (NG_011798.1: g.54253C>A) and between Exon_31 and Exon_32 (rs17216212).

The allele frequency of the alleles in the population studied in the central savannah was compared to the frequency reported in the project 1000 G and 1000 G Colombia for the Population in Medellín. For genes expressing the MRP1 protein, 0 of the 14 polymorphisms were involved in the transition G to A. For the SNPs which have been referenced to and were located in the intron between exons_2 and _3 (Table 3), rs200647436 was found to be in HW equilibrium (*p* = 0.4) in the study population; however, when compared with those reported in other populations, a greater proportion of allele A is found in the Central Savanah although it is still the lowest population allele in all populations. For the rs2000624910 compared to CLISEQ_SNP, a significant value of *p* is observed, indicating a lower proportion of allele C in the sample. For new SNPs identified in MRP1/ABCC1, an HW equilibrium is observed with *p* > 0.05, except for polymorphism NG_028268.1:g.138867G>A (*p* < 0.05). HW could not be determined for SNP rs150214567 because the entire study population was homozygous C/C, with no differences in population frequencies with the 1000 genomes. Therefore, the new SNPs identified in the study could not be compared to other population data.

Polymorphisms referenced for MRP2/ABCC2 exons can be observed in Table 4. The SNPs rs2273697 and rs3740066 are found to be in HW equilibrium (*p* = 0.5 and *p* = 0.24), but their population frequency differs significantly from other populations. For the SNP rs17216212 and rs142573385, an HW imbalance with *p* < 0.05 was observed. Interestingly, for SNP rs3740066, the genotype count had only one homozygous behavior (T/T) with an allelic frequency significantly different to the world population (*p* = 0.01) and the population of Medellín (*p* = 0.01). Finally, there was no difference in frequency for the SNP rs142573385 when compared to the world population, which is contrary to that reported in 1000 G Colombia (*p* = 0.04); a significant difference was observed for both global frequencies and the population of Medellín in the case of SNP rs17216212. There is an HW equilibrium with a *p* = 0.07 in the case of NG_011798.1:g.41261_A. It was not possible to determine the full allele frequencies for SNP NG_011798.1:g.54253A>C as it was not possible to analyze the electropherograms for this exon (Ex_23) despite having had the samples re-sequenced.

## 4. Discussion

Exons studied for MRP1/ABCC1, and MRP2/ABCC2 genes had been previously reported in other studies [22,29,30], which indicated the presence of polymorphisms both in exons and intron regions. The decision to sequence exons is due to more significant evidence of the functional relationship between genetic variants in coding regions and their clinical consequences, especially drug adverse events [29,31,32]. Most polymorphisms found in the research were not found in the exonic regions, which suggests they are located within primary mRNA (transcripts containing introns) as secondary mRNA (transcripts which no longer contain introns). The previous information includes untranslated regions like 5′UTR and 3′UTR, intron, and coding regions (non-synonymous) that could affect the structure and function of RNA and, therefore, the functionality of proteins, the regulation of translation of mRNA protein, and mRNA polyadenylation [33]. Additionally, the presence of SNPs in intronic regions could affect the expression or function of the proteins with an alteration of the stability of the mRNA affecting splicing, translational control, or regulation. Polymorphisms in the promoter region 5′UTR and 3′UTR are known to influence the promoter activity and, therefore, the expression or stability of the mRNA transcribed [29]. Some polymorphisms are involved in gene expression, which depends on cis and trans-regulatory elements located in the promoter regions of genes that may alter, destroy, or create binding sites and recognition of transcription factors, alter the levels of gene expression or may lead to a subexpression thereof [33]. Pratt found new variants of ABCC2 haplotypes in cis on the chromosome of individuals (rs17222723 and rs8187718) [34].

A search of SNPs reported in dbSNP [35] and published studies [25,36,37] revealed that two SNPs, located in the protein MRP2/ABCC2 (rs2273697 and rs3740066) and found in our study, had been previously reported with clinical implications. One of these implications is associated with the resistance to antiepileptic drugs in the Asian population. The overexpression of ABCC2 primarily in endothelial cells of the hematoencephalic barrier, liver, intestine, kidney, placenta, and lungs has been reported to be a risk factor for the resistance to drugs used in epilepsy, cancer, and hepatitis [38,39]. Previous studies have found a significant association of rs2273697 and rs3740066 with resistance to antiepileptic drugs in Caucasian and Chinese patients [25,26,30]. Additionally, rs2273697 in disequilibrium with rs717620 haplotype increases the risk of resistance Carbamazepine (CBZ) [18]. Similar data have shown the presence of rs3740066>66744 T>G in the Mexican population, which has been identified as a high-risk factor for drug resistance to antiepileptic drugs [36]. On the other hand, polymorphism rs2273697 1249 G>A was studied in a Japanese population to evaluate the proximal kidney tubular dysfunction (KTD) with Tenofovir and reported an association of this genotype with KTD [37].

Interestingly, we found a polymorphism of the gene encoding MRP2/ABCC2 given by the presence of AG genotypes SNPs rs2273697 in position 1249 in the study population (i.e., 8 volunteers out of 50). This finding indicates a protective factor for tubular renal dysfunction in patients with HIV taking Tenofovir as reported in the literature [40]. The novel polymorphisms which had not been previously reported and were found in our population open up a possibility to continue to study them in patients under treatment with drugs associated with these proteins and to determine an association between the SNP and the response to treatment. It is noteworthy to mention that some of these polymorphisms are found in different proportions in this sample of the Colombian population, as compared to those reported in 1000 G. Interestingly, the presence of polymorphisms where the genotype count is fully homozygous (rs3740066 and rs2273697) is striking, given that an endogamous population was not studied, thus differentiating them from other populations described. This study projects a second stage in which the frequencies of SNPs are to be validated including different populations in Colombia. However, even if the frequencies are different in future validation studies, it is essential to identify individuals with homozygous variants of the minor allele, since these are responsible for the most significant risk of failure of treatments; that is, regardless of the population frequency, the identification of a single individual whose genotype has clinical interest is sufficient to comply with the principles of personalized medicine and the role of pharmacogenetics in clinical care.

New in silico studies can help establish criteria for studies on these mutations so as to determine whether the presence of various SNPs may have a functional effect, that is, an additive effect, or whether the new SNP in the homozygous state has some sort of advantage over the metabolism of toxins or other endogenous factors. Additionally, those volunteers in our study with the most substantial number of polymorphisms can be considered for further analysis with other regions of the encoded proteins by genes MRP1/ABCC1 and MRP2/ABCC2 supplemented with new population analysis. On the other hand, it is possible to analyze SNPs identified from an epigenetic point of view.

Study limitations need to be recognized. First of all, not all exons were studied for MRP1/MRP2 ABCC1 and ABCC2 proteins. However, the data provided in this study opens a perspective on the presence of these polymorphisms in this population and the possibility for future clinical studies evaluating clinically these transporter proteins.

Statistical differences, although significant in the present study, may change with regards to larger population samples. However, the presence of polymorphisms in well-selected populations is part of the necessary characterization of genetic variants that have potential clinical significance. Regarding sample size, the studies of population characterization of genetic variants fluctuate, even if those studies imply characterization of individuals or tissue samples [36,41]. Moreover, the reference population, as it is 1000 genomes, varies as well, giving us an only a representation of particular communities that can be biased for several reasons, including selection bias and inadequate representation of population stratification [42]. However, this is a descriptive study, not a genetic association study. Therefore, it must lead to future replications in large samples, and its interpretation must consider clinical contexts and not only frequencies of the genetic variants [43].

## 5. Conclusions

In our study, several polymorphisms in MRP1/MRP2 ABCC1 and ABCC2 were studied. The functional properties of these polymorphisms are still unknown in our population. Future studies are necessary to identify whether the presence of these SNPs modifies the expression of this protein family, and thus the function thereof. However, the results of this study provide valuable information to establish the basis of individualized drug therapy. On the other hand, new research studies are projected of populations, in order to determine a frequency validation on a larger scale and to establish the reason why the population studied differs in some cases from the global population and from the population of Medellín reported in 1000 genomes.

## Figures and Tables

**Table 1:** The pharmacological substrates and inhibitors of multidrug resistance-associated proteins (MRP) 1 and 2 mediating drug resistance [16,20,27,28].

Gene/Protein	Substrates	Inhibitors	Consequences of Mutation/Polymorphism
MRP1 (ABCC1)	Various glutathione, glucuronide, and sulfate conjugates, anthracyclines, vinca alkaloids, etoposide, teniposide, topotecan, SN-38, melphalan, methotrexate, non-organic heavy metal oxyanions, leukotriene C4 (LTC_4_), D4, E4.	Sulfinpyrazone, probenecid, MK571, LTC4, some Pgp inhibitors (e.g., cyclosporin A, verapamil, PSC 833)	Reduction in intracellular concentration of the drug reduced clearance. Association with resistance in cancer and infectious diseases
MRP2 (ABCC2)	bilirubin, cisplatin, pravastatin, sulforhodamine 101 acid chloride (Texas Red), GSH, GSH conjugates, glucuronide conjugates, Olmesartan, valsartan, vinblastine, vincristine, etoposide, methotrexate, cisplatin, rifampicin, sulfinpyrazone, ceftriaxone, camptothecins, mitoxantrone, saquinavir	MK571, furosemide, probenecid, protease inhibitor (ritonavir, saquinavir), nucleoside analog (lamivudine, abacavir, emtricitabine), nucleoside phosphonate (cidofovir, adefovir, tenofovir)	Reduced expulsion of bilirubin. Rapid decrease of substrate bioavailability and increased biliary excretion

**Table 2:** The primers for the sequence amplification of MRP.

Gene/Protein	Exon	Primer Direction	Longitude (bp)	Sequence (5′-3′)	Longitude Amplicon (bp)
MRP1 (ABCC1) [29]	2	Forward	20	GCAGAAGACACCACATACCT	510
		Reverse	20	AGAAGAAGGAACTTAGGGTC	
	10	Forward	18	TCCTGGGCAGACAGATAG	439
		Reverse	18	TGAACCACAGCCGGAACT	
	16	Forward	20	GTTTAGTACAGTCTTGCCTT	463
		Reverse	19	CCAAAATCCTGCCTTCTAG	
	17	Forward	21	GTGGGCCAGCTGTTGTCTCGT	441
		Reverse	20	AGTGAGACCTGAGCCACACC	
	23	Forward	20	ATGCCTGGTTCATCATTATT	514
		Reverse	20	CTTTAGGTAACACTGGTATA	
MRP2 (ABCC2) [23]	10	Forward	21	GGGTCCTAATTTCAATCCTTA	310
		Reverse	21	TATTCTTCTGGGTGACTTTTT	
	18	Forward	21	GGAGTAGTGCTTAATATGAAT	249
		Reverse	21	CCCACCCCACCTTTATATCTT	
	23	Forward	21	TGCATGGTGCTGACAAAACTG	218
		Reverse	21	CACCACCTGACAGTTCTTGAG	
	28	Forward	21	TGCTACCCTTCTCCTGTTCTA	269
		Reverse	21	ATCCAGGCCTTCCTTCACTCC	
	31	Forward	21	AGGAGCTAACACATGGTTGCT	272
		Reverse	21	GGGTTAAGCCATCCGTGTCAA	

bp: base pair.

**Table 3:** The polymorphism ABCC1 ATP-binding cassette, sub-family C (Cystic Fibrosis Transmembrane Conductance Regulator (CFTR)/MRP), member 1.

Location	Polymorphism (*n*)	Allelic Count		Genotypic Count			HW ^1^	1000 G ^2^	1000 G Colombia ^3^
After Exon 2	rs200647436 (45)	A	G	A/G	G/G		*p*-value	*p*-value	*p*
		10(11.1%)	80(88.9%)	10	35		0.4	0	0.000003
	NG_028268.1:g.63440G>A (45)	A	G	A/G	G/G		*p*-value	*p*-value	*p*
		1(1.11%)	89(98.8%)	1	44		0.93	-	-
	NG_028268.1:g.63452G>A (45)	A	G	A/G	G/G		*p*-value	*p*-value	*p*
		85(94.4%)	5 (5.5%)	5	40		0.69	-	-
	rs200624910 (45)	C	G	C/G	G/G		*p*-value	CLINSEQ_SNP ^4^ *p*	
		2(2.22%)	88(97.7%)	2	43		0.87	0.002	
rs200647436: Count allelic 1000 Genomes: 4 (A)/5004 (G), Colombia allelic count: 188 (G). rs200624910: allelic CLINSEQ_SNP Count 3 (C)/1320 (G). NG_028268.1: g.63440G>A and NG_028268.1: g.63452G>A not been reported previously.									
After Exon 10	NG_028268.1:g.103730T>G (41)	G	T	T/G	T/T		*p*-value	*p*-value	*p*-value
		1(2.4%)	81(98.8%)	1	40		0.94	-	-
NG_028268.1:g.103730T>G has not been reported previously.									
After Exon 16	NG_028268.1:g.134922G>A (45)	A	G	A/G	G/G		*p*-value	*p*-value	*p*-value
		2(2.22%)	88(97.78%)	2	43		0.87	-	-
	NG_028268.1:g.134927G>A (44)	A	G	A/G	G/G		*p*-value	*p*-value	*p*-value
		2(2.27%)	86(97.73%)	2	42		0.87	-	-
	NG_028268.1:g.134935G>A (44)	A	G	A/G	G/G		*p*-value	*p*-value	*p*-value
		1(1.14%)	87(98.86%)	1	43		0.93	-	-
	NG_028268.1:g.134940G>A (44)	A	G	A/G	G/G		*p*-value	*p*-value	*p*-value
		1(1.14%)	87(98.86%)	1	43		0.93	-	-
	NG_028268.1:g.134949T>A (44)	A	T	A/T	T/T		*p*-value	*p*-value	*p*-value
		1(1.14%)	87(98.86%)	1	43		0.93	-	-
None of the polymorphisms has been reported previously.									
Exon 17	NG_028268.1:g.138867G>A (46)	A	G	A/G	G/G	A/A	*p*-value	*p*-value	*p*-value
		5(5.43%)	87(94.57%)	1	43	2	0	-	-
	NG_028268.1:g.138902C>A (46)	A	C	A/C	C/C	A/A	*p*-value	*p*-value	*p*-value
		1(1.09%)	91(98.91%)	1	45	0	0.94	-	-
After Exon 17	NG_028268.1:g.139009C>G (46)	C	G	C/G	G/G	C/C	*p*-value	*p*-value	*p*-value
		0(0%)	46(100%)	0	46	0	-	-	-
None of the polymorphisms have been reported previously.									
Before Exon 23	rs150214567 (43)	T	C	T/C	C/C	T/T	*p*-value	*p*-value	*p*-value
		0(0%)	86(100%)	0	43	0	-	0.85	0.5
rs150214567: 1000 Genomes allelic count: 2 (T)/5006 (C), Colombia allelic count: 188 (C).									

^1^ HW: Hardy Weinberg. AA = p^2^, AB = 2pq, BB = q^2^. A = G, B = A. Population: *H*_0_: The allele frequencies are the same in both populations. *H*_1_: The allele frequencies are different in both populations. Significance of *p* < 0.05. ^2^ Allele frequencies 1000 Genomes. ^3^ Allele frequencies in 1000 Genomes—Colombian Population (Medellín). Population data from the National Human Genome Research Institute (NHGR). Introns outside the region NG02826.

**Table 4:** The polymorphism ABCC2 ATP-binding cassette, sub-family C (CFTR/MRP), member 2.

Location	Polymorphism (*n*)	Allelic Count		Genotypic Count			HW ^1^	1000 G ^2^	1000 G Colombia ^3^
Exon 10	rs2273697 (45)	A	G	A/G	G/G	A/A	*p*	*p*	*p*
		8(8.89%)	82(91.11%)	8	37	0	0.5	0.018	0.13
rs2273697: 1000 Genomes allelic count: 934 (A)/4074 (G), Colombia allelic count: 29 (A)/159 (G)									
After Exon 18	NG_011798.1:g.41261_A (28)	T	A	A/T	A/A	T/T	*p*	*p*	*p*
		14(25%)	42(75%)	14	14	0	0.07	-	-
NG_011798.1:g.41261_A Has not been reported previously. In 13 individuals it was not possible to clearly determine the genotype. It is not possible to determine the full allele frequencies for forty individuals.									
Before Exon 23	NG_011798.1:g.54253A>C (47)	A	C	A/C	A/A	C/C	*p*	*p*	*p*
		I	I	I	I	I	I	-	-
NG_011798.1: g.54253A>C has not been reported previously. 18 subjects in genotype C/C was determined. In 29 subjects could not clearly establish the genotype. It is not possible to determine the full allele frequencies.									
Exon 28	rs3740066 (49)	T	C	T/C	C/C	T/T	*p*	*p*	*p*
		84(85.71%)	14(24.5%)	14	0	35	0.24	0.01	0.01
rs3740066: 1000 Genomes allelic count: 3565 (C)/1443 (T). Colombia allelic count: 121 (C)/67 (T).									
Exon 31	rs142573385 (45)	T	C	T/C	C/C	T/T	*p*	*p*	*p*
		2(2.22%)	88(97.78%)	0	44	1	0	0	0.04
rs142573385: 1000 Genomes allelic count: 5006 (C)/2 (T). Colombia allelic count: 188 (C).									
After Exon 31	rs17216212 (43)	A (%)	G (%)	A/G	G/G		*p*	*p*	*p*
		-	86(100%)	-	43		0	0.06	0.12
rs17216212: 1000 Genomes allelic count: 191 (A)/4817 (G).									

^1^ HW: Hardy Weinberg. AA = p^2^, AB = 2pq, BB = q^2^. A = G, B = A. Population: *H*_0_: Allele frequencies are the same in both populations. *H*_1_: Allele frequencies are different in both populations. Significance of *p* < 0.05. ^2^ 1000 allele frequencies in Genomes. ^3^ Allele frequencies in 1000 Genomes—Colombian Population (Medellín). Population data from National Human Genome Research Institute (NHGR). Introns outside the region NM_000392.4.
